# Hospital diet for COVID-19, an acute respiratory infectious disease: An evidence-based Protocol of a Clinical Trial

**DOI:** 10.22088/cjim.11.0.466

**Published:** 2020

**Authors:** Masoud Moslemifard, Narges gorji, Reza Ghadimi, Mohammad Kamalinejad, Hoda Shirafkan, Seyyed Ali Mozaffarpur

**Affiliations:** 1 Department of Persian Medicine, School of Persian Medicine, Babol University of Medical Sciences, Babol, Iran; 2 Department of History of Medical Sciences, School of Persian Medicine, Babol University of Medical Sciences, Babol, Iran; 3 Social Determinants of Health Research Center, Research Health Institute, Babol University of Medical Sciences, , Babol, Iran; 4 School of Pharmacy, Shahid Beheshti University of Medical Sciences, Tehran, Iran; 5 Traditional Medicine and History of Medical Sciences, Research Health Institute, Babol University of Medical Sciences, ,Babol, Iran

**Keywords:** Persian medicine, Herbal Medicine, COVID-19, diet, Medicine, Traditional, Coronavirus

## Abstract

**Background:**

COVID-19 caused a global pandemic problem. No confident management is introduced for it yet. This study aimed to propose a dietary protocol for hospitalized patients with the diagnosis of acute respiratory infectious disease caused by COVID-19 based on Persian Medicine.

**Methods:**

This study was conducted in three phases. In the first phase, any diseases that could be matched with the clinical features of infection with COVID-19 were searched in selected PM references. In the second phase, medicinal herbs and foods that were available and could be used in the hospital diet were extracted and summarized. In the third phase, the new documentation of these pharmaceutical and food items was conducted.

**Results:**

The signs and symptoms of infectious respiratory disease caused by COVID-19 can be categorized in the field of Zato al-rieh that can mainly be matched with pneumonia. Based on the described criteria, some nutrients and medicinal materia medica have been introduced for acute respiratory infection including Cydonia oblonga, Honey, Citrus sinensis, Malus domestica, Citrus medica, Crocus sativus, Raisin, Rosa Damas Cena, D.Carota, Camellia Sinensis, Anethum graveolens dhi, Punica granatum, Petroselinum Crispum, Coriandrum sativum, Urtica dioica, Allium sativum, Sesamum indicum

**Conclusion:**

Most materia medica has documents in current articles including anti-cough suppressants, antiviral properties, anti-bacterial, anti-inflammatory, antioxidant, immunomodulatory etc. A protocol of hospital diet for patients with infectious respiratory syndrome caused by COVID-19 has been introduced in this manuscript.

Coronaviruses can cause infectious respiratory diseases with signs and symptoms from a simple common cold to SARS, MERS and COVID-19. COVID-19 was identified in Wuhan, China in December 2019, and quickly spread to many countries and became a global pandemic problem (1, 2). Clinical manifestations of respiratory infectious diseases including fever, dry cough, shortness of breath, weakness and lethargy and myalgia are the key symptoms of the disease. Also loss of appetite, nausea, diarrhea and increase in liver enzymes occurred in some cases (3). The period between exposure to infection is 3-7 days and sometimes up to 14 days and some studies mentioned up to 18 days (4) COVID-19 disease is mild and self-limiting in most patients, but in some, especially those with comorbidities, is severe and can lead to death (5, 6). As any drug of choice has not been introduced for COVID-19 yet, seeking ideas in traditional medicine references is a logical way (7, 8). It has been proposed that early initiation of complementary medicine recommendations can reduce the duration and severity of the disease (9).

Based on traditional Chinese Medicine (TCM), similarities with different syndromes of TCM have been expressed in different stages of COVID-19 (1). Also, based on TCM some relationships between coldness and wetness of environments with the prevalence of infection were introduced (9). In another study in TCM, the presence of fever, dry cough and shortness of breath in COVID-19 disease were considered as equivalent of increased heat and dryness (1). Persian Medicine (PM), with a long history has been presented with new evidences (10-13). Many documents in PM references have been recorded about the epidemy of infectious diseases and how to deal with it (14-16). According to the basic theories of PM, during the epidemy of infectious diseases, people with cold and wet prevalence (dominance of phlegm) are more prone to infection than others, and therefore it is recommended to provide warm-hearted preventive recommendations. As a number of patients with COVID-19 require hospitalization, providing a diet that along with other supportive therapies can help the patient to reduce hospitalization as well as mortality can be beneficial.

This study aimed to review the references of PM to propose a dietary protocol for hospitalized patients with the diagnosis of acute respiratory infectious disease caused by COVID-19. 

## Method

This study was conducted in three phases. In the first phase, the clinical manifestations of COVID-19including fever, shortness of breath and cough were searched in the selected PM references. The names of theses texts were Ferdows al-hekmah fi al-tibb (17), Qanun fi al-Tib (18), Mansouri fi al-Tib (19), Kamel al-Sanaah al-Tibbiyah (20) Zakhireye Kharazm Shahi (21), Hedayat al-mota’allemin fi al-tibb (22), al-Advieh va al-Aghzieh(23), Al-Shamel (24), Konnash fi al-Tib (25), Sharh e al-Moujaz (26), Menhaj al-Dokan (27), Aqhili treatments (28), Jame fi al-Advieh va al-Aghzieh (23), Exir Azam medicine (29), Tohf al-Momenin (30) and the Makhzan-al Advieh (31). The texts related to these diseases were extracted from the reference.

In the second phase, medicinal herbs and foods that were available and could be used in the hospital diet were extracted and summarized.In the third phase, the new documentation of these pharmaceutics and food items was searched in databanks including Web of science, PubMed, Scopus, science direct, SID and Google scholar. Finally, these findings are presented as a usable treatment protocol. This study with the code of MUBabol.HRI.REC.1399.104 was approved by the Ethics Committee of Babol University of Medical Sciences.

## Results

**Diseases corresponding to COVID-19 in PM references: **In PM, the signs and symptoms including fever and shortness of breath accompanied by cough were explained in several respiratory diseases. Some of these diseases could be prevalent as a pandemic infection in short time (18, 20, 32-34). *Rhazes *(854–925 AC) in his books *Al-Havi Fi Al-Tib* and *Al-Mansouri Fi Al-Tib* has pointed out the term "pneumonia (*Zato al rieh*)" as an acute respiratory syndrome (19). *Hakim Azam Khan* (died in 1902 AC) in his book *"Eixir e Aazam"*, referring to the majority of physicians, considers pneumonia to be swelling in lung which can be transmitted from a patient to a healthy person (29). *Avicenna* (980-1037 AC) describes the signs and symptoms of pneumonia including fever, thirst, cough, shortness of breath, feeling of heaviness and stretching in the chest, and pain between two shoulders, which in some cases lead to pleural effusion or death because of the heart involvement (18). According to PM viewpoints, it seems the characteristics of pneumonia as an acute transmitted respiratory infection with fever; cough and shortness of breath were similar to Covid-19 manifestations.

**General advice for acute respiratory syndrome: **The general advice in managing acute respiratory syndrome is to use foods and herbal medicines that reduce fever and improving respiratory function. Based on the principles of PM, the composition of the diet appropriate for respiratory infection similar to COVID-19 should be with low amount and high nutritional value for strengthening the lung and whole body.

Medicinal herbs proposed in PM references: Based on the described criteria, some nutrients and medicinal materia medica have been introduced for acute respiratory infection including Cydonia oblonga, Honey, Citrus sinensis, Malus domestica, Citrus medica, Crocus sativus, Raisin, Rosa Damas Cena, D.Carota, Camellia Sinensis, Anethum graveolens dhi, Punica granatum, Petroselinum Crispum, Coriandrum sativum, Urtica dioica, Allium sativum, Sesamum indicum. In most of these materia medica documents of therapeutic effect in infections of respiratory tract have been found. The details of founded materia medica in PM references and current medicine is shown in Table 1. 

**Table1:** Materia medica proposed in in PM references for treatment of acute respiratory infectious syndrome

**Documents in articles**	**Indications in PM references**	**Part of materia**	**common name in PM**	**common name**	**scientific name**
Antioxidant activity Anti-inflammatory Antimicrobial activities(35) anti-influenza viral activity(36)	‏ Beneficial for lung(17) Softening agent(23) Anti-cough(37) Anti-hoarseness (31) Advise in dyspnea and any swelling in lung(21)	fruit	Beh	Quince	Cydonia oblonga
Anti-cough Anti-inflammatory Antioxidant, Immunomodulatory Antiviral and anti-Bacterial (38-40)	Anti-sore throat, anti-cough (21, 22, 30, 31)	-	Honey	Honey	Honey
Anti-viral Antioxidant(41, 42)	Anti-hoarseness(31)	fruit	Konleh Portoghal	Sweat orang	Citrus sinensis
Anti-influenza Antioxidant and antimicrobial properties(43, 44) Anti –asthma(45)	Effective in dyspnea and shortness of breath Effective in pleurisy, fever and cough(29)	fruit	Sib	Apple	Malus domestica
Analgesic Antimicrobial Antioxidant Anti-inflammatory(46)	Effective in airborne diseases(31)	fruit	Balang	A kind of citrus	Citrus medica
Antioxidant properties(47) Anti-inflammatory(47-49) Anti- viral(50) Antidepressant(51)	Antidepressant, protective against airborne diseases(29)	flower	Zaferan	Saffron	Crocus sativus
Anti-oxidants(52-54) Antibacterial(54)	Anti-cough(29)‏	fruit Raisin	Keshmesh	Grape	Vitis vinifera.
Cough suppressant(55) Respiratory smooth muscle relaxant(56) Anti-HIV Activity(57) Antioxidant, Antibacterial Activities(58) Anti-inflammatory and analgesic(59)	Improving respiratory function, effective in hemoptysis(29)‏	Distillate leaf	Golab	Damask rose	Rosa Damas Cena
Anti-bacterial Anti-fungal Anti-oxidants Anti-inflammatory Analgesic(60)	Anti-cough, decreasing the secretion of trachea an sinuses(30)(25),	root	Jazar Havij	Carrot	Daucus carota
Antimicrobial and Antiviral Activity(61) Analgesic, Antipyretic, Anti-Allergic Activity, and Anti-Inflammatory (62) Antioxidant(63)	Diluting lung mucosa, effective in dyspnea, effective in shortness of breath(31) Decreasing post nasal drainage, effective in shortness of breath(29)	leaf	chay	Tea	Camellia Sinensis
Antiviral(64) Anti-inflammatory, Analgesic(65) Antioxidant(66) Antibacterial, Antifungal(67)	Effective in Asthma and dyspnea(17, 20)‏ Improving respiration,effective in Asthma (29, 231) Effective in pleurisy(25)	leaf	Shevid	Dill	Anethum graveolens dhi
Antioxidant(68) Anti-inflammatory effect(69)	Anti-cough(17, 31) (20) Anti-hoarseness (20) ‏ Diluting the lung secretion, Anti-fever (25)(20)	Fruit extract	Anar	Pomegranate	Punica granatum
Anti-oxidant activity(70) Anti-Inflammatory Activity(71) Analgesic and spasmolytic activity(72) Immunomodulating activity(73)	Diluting the lung secretion, Anti-cough (31)	leaf	Jafary	Parsley	Petroselinum Crispum
Antiviral(74) Antibacterial activity(75) Antifungal activity(76) Antioxidant activity(77) Food preservation and anti-spoilage(78)	Effective in hemoptesis(19)‏ Anti-cough and dyspnea(31)	Leaf seed	Geshniz	Coriander	Coriandrum sativum
Antiviral activity(79, 80) Antibacterial, antioxidant, analgesic, anti‑inflammatory, immunomodulatory(81, 82)	Diluting the lung secretion, effective in Asthma and dyspnea (with the source of lung)(31)‏ Effective in most kinds of lung diseases(24) Effective in Asthma(29)	leaf	Gazaneh	Nettles	Urtica dioica
Antiviral(83-85) Antioxidant, Antimicrobial activity(86) Cancer prevention(87)	Decreasing the secretion of the lungs, anti-asthma and dyspnea(31)‏ Anti-hoarseness and cough(24)	pulp	Sir	Garlic	Allium sativum
Antioxidant, antimicrobial(88), anti-inflammatory(89), hepatoprotective(90),	Anti-hoarseness and cough )^24^, ^31^) ‏	seed	Konjed	Sesame	Sesamum indicum

## Discussion

The growing trend to traditional medicine treatments worldwide, including Iran in recent decades, has shown the responsibility and importance of research in this field more than ever. PM as one the most important paradigms of traditional medicine focus of lifestyle modification for maintaining health and management of diseases. While there is not any introduction of any effective drug in controlling COVID-19 in hospitalized patients, proposing a diet may help as a supportive role in controlling the disease.Clinical and therapeutic experience of COVID-19 showed that the early intervention of traditional and complementary medicine along with common therapies leads to shortening the disease period and delaying diseaseadvancement and reduces mortality rate. Materia medica has been proposed in this study for managing infectious respiratory disease caused by COVID-19 has documents in new studies that make their effects on the COVID-19 imaginable.

The quince fruit with the scientific name of Cydonia oblonga is rich in mineral elements (iron, phosphorus, calcium, magnesium, and potassium), organic acids (malic acid, ascorbic acid, aspartic acid, citric acid, and glutamic acid), fiber, carbohydrates, amino acids, vitamins, and tannins (91). Flavonoids and phenolic acids in quince contribute with antimicrobial activities, antioxidant capacity, anti-carcinogenic, anti-inflammatory, anti-ulcerative activities, anti-allergic effects (35).

Effect of **honey** was investigated in randomized double-blind study in children with common cold in treatment of cough, comparing with salbutamol and placebo. The results showed honey was the most effective in symptomatic relief of symptoms associated with the common cold whilst salbutamol or placebo offered no benefits (38). Another clinical study compared the effect of honey with dextromethorphan, and no treatment on nocturnal cough and quality of sleep in children and their parents. The results showed that honey was the most effective in a child's nocturnal cough and sleeping difficulty was caused by upper respiratory tract infection (39).

The results of a systematic review and meta-analysis about the effectiveness of honey for symptomatic relief in upper respiratory tract infections showed superiority of honey comparing with the usual care for the upper respiratory tract infection symptoms. It could help efforts to slow the spread of antimicrobial resistance and provide a widely available and cheap alternative to antibiotics (40).

**Citrus sinensis** (sweet orange) essential oil (EO) showedstrong anxiolytic and good radical-scavenging activity (41, 42). EO of sweet orange can inhibit the growth of several bacteria including Listeria monocytogenes, Staphylococcus aureus, Salmonella typhimurium, Vibrio parahaemolyticus, Escherichia coli, and Pseudomonas aeruginosa (92), as well as several fungal species, such as Aspergillus flavus, A. fumigatus, A. Niger, and Trichoderma viride (92). In addition, an intense larvicidal activity against the malaria vector, Anopheles labranchiae (93) and the vector of yellow and dengue fever, Aedes aegypti (94) have been reported. Inhalation of orange EO increases comfortable, relaxed, and natural feelings with a significant decrease in oxyhemoglobin concentration in the right prefrontal brain cortex (95). The odor of sweet orange improves the mood and decreases the symptoms of anxiety (96). 

**Malus domestica**, due to their flavor and sweetness is one of the most consumed fruits in the world (43). Generally, flavonoid intake is associated with a lower risk of asthma, and is attributed mainly to quercetin, hesperitin, and naringenin (97). Previously apple consumption has been inversely linked with asthma. It has been positively associated with general pulmonary health. It has shown in a recent study that apple and pear intake has been associated with a decreased risk of asthma and a decrease in bronchial hypersensitivity in 1600 adults, in Australia (45). Also, it was reported in adults in the United Kingdom, as selenium intake, apple intake was associated with less asthma (44). A reduced risk of cardiovascular disease has been associated with apple consumption especially in women (98).

**Citrus medica** Linn is widely used in traditional system of medicine. Citrus medica Linn. Possesses hypoglycaemic, analgesic, anticholinesterase, antidiabetic, hypocholesterolemic, anticancer, hypolipidemic, insulin Secretagogue, anthelmintic, antimicrobial antiulcer and estrogenic properties. The mechanisms of these effects are not completely understood (48). 

Saffron (**Crocus sativus** L.) grows mainly in Iran, Kashmir (India and Pakistan), Azerbaijan, Turkey, China, Morocco, Spain, Libya, Greece, Mexico, and Austria. Crocin (a water-soluble yellow and active component), crocetin, safranal are the main components of saffron (49). In addition, saffron includes sugar, protein, amino acids, flavonoids, vitamins, and vital minerals. Although saffron includes more than 150 volatile and aromatic compounds such as lycopene, zeaxanthin, α- and β-carotene that its golden and orange color is caused by the α-crocin (53). In some researches anti-inflammatory, antioxidant, and immunomodulatory properties of Crocus sativus have been recommended (47, 49). It is reported that saffron can benefit all organs and health problems including asthma, bronchitis, coughing, nervous systemheart health, teeth and gums, and eyes (48). It has also an anti-viral effect (50). Because of its antioxidant effect as well as anti-inflammatory, hypolipidemic, and anti-carcinogenic effects, saffron is used as a natural agent for the treatment of diseases in traditional medicine (47).

Raisins (**Vitis vinifera**.) in both in vitro and in vivo experiments, showed excellent antioxidant capacity (36). The content of phenolic compounds cause the antibacterial and antioxidant activity of raisins (52). Many studies proposed a positive correlation between the total phenol content in raisins and antioxidant capacity (53). Specific polyphenols such as procyanidins, catechinand, quercetin have been correlated with its antimicrobial and antioxidant activities (54).

An ornamental plant **Rosa Damascena** had been used from ancient times to cure many infectious disorders. It has excellent natural potential against inflammatory diseases. Juice from fresh rose petals contains outstanding antioxidant properties (99). This plant contains flavonoids such as quercetin and caempferol plus their glycoside derivatives, myrcene, terpene, tannins, carboxylic acids and vitamin C (100). In one study, the effect of ethanolic, aqueous and chlorphormic extracts in mice on hot plate and tail flick was studied. Its ethanolic extract showed analgesic effect (101). Research about respiratory effect of R. dama scena is sparse and one animal study evaluated the guinea pigs, showed that the ethanolic and aqueous extracts of this plant significantly reduced severity of coughs induced by citric acid (102). In another animal study in guinea pigs, the effect of its ethanolic extract and essential oil on tracheal smooth muscle showed a potent relaxant effect of extract and essential oil could be comparable with theophylline (56). Also anti-HIV activity (57), antioxidant, antibacterial activities (58) and anti inflammatory (59) effects have been suggested for Rosa damascena in animal studies

Carrot (**Daucus carota** subsp. Sativus) is a root vegetable with carotenoids, polyacetylenes, flavonoids, vitamins, and minerals. Besides the old belief that carrots are good for the eyes, polyphenols, carotenoids, and vitamins present in carrots effect as anticarcinogens, anti-oxidants, and immune enhancers. Anti-diabetic, anti-hypertensive, hepatoprotective, reno protective, cholesterol and cardiovascular disease lowering, and wound healing benefits of carrots have been reported. The anti-inflammatory, cardio- and hepatoprotective, anti-fungal, anti-bacterial, and analgesic effects of carrot seed extracts are also noteworthy (60).

Green tea (**Camellia Sinensis) **is considered a reference drug with respect to its anti-oxidant potency, in traditional medicines (63). This effect may be attributed to the polyphenols that are represented by its flavanols, which can be readily oxidized to the corresponding o-quinones, and cause a function as hydrogen acceptors, as well as hydrogen donors (103). The interaction with reactive oxygen species could be done by the polyphenolic compounds in green tea by possessing different degrees of free radical scavenging properties, particularly towards oxygen-free radicals and to some extent towards nitrogen (NO) species’ production inhibition (104).

Some studies’ antimicrobial activity of green tea has been reported. Green tea is effective against Staphylococcus aureus, Staphylococcus epidermidis, and Vibrio cholerae O1 and also effective against bacteria that cause dental caries, including Streptococcus salivarius, Escherichia coli, and Streptococcus mutans (61). Also, a potent anti-inflammatory and antipyretic effect of green tea has been reported (62). 

Dill (**Anethum graveolens**) that belongs to the Umbelliferae family is an annual or biennial herb. Evidence showed that consumption of dill leaf could provide good antioxidant activities (65, 66, 105, 106).The hydro alcoholic extract of Anethum graveolens shows reduction in inflammation (65) and pain in rats (107). The analgesic effect has shown 10 % aqueous extract of fruit and 5% aqueous solution of essential oils in mice (induction of pain by hot plate and acetic acid writhing methods (108). Dill tablet presented significant DPPH radical scavenging activity due to the amounts of phenolic content (109). Also, antifungal (67) and anti-viral effects (64) of Dill have been proposed.

The seed oil and juice of Pomegranate** (Punica granatum**) contains some kinds of flavonoids and anthocyanidins (cyaniding, delphinidin and pelargonidin) that can be beneficial for the prevention of inflammatory, cardiovascular and other diseases. These components prevent oxidative stress that induces lipid peroxidation in lipoproteins and in arterial macrophages (66). Punica granatum inhibits Cyclooxygenase (COX) and lipooxygenase (LOX), the key enzymes in the conversion of arachidonic acid to prostaglandins and leukotrienes (important inflammatory mediators), respectively (69).

Including leavesof** Petroselinum crispum** to the diet caused significant increase in antioxidant enzymes comparing with the basic diet received group (70). It was demonstrated that Apigenin supposedly the main compound is responsible for this activity (110). In various in vitro models, different extracts of Petroselinum crispum leaves and stems exhibited antioxidant properties (111). Also, the hydroalcoholic extract of Petroselinum crispum seed revealed analgesic activity in mice (72)(56). Also, essential oil from Petroselinum crispum seed suppressed humoral and cellular immune response via inhibiting splenocytesand macrophages function (73). In some studies the antioxidant, anti-bacterial (70) and anti-inflammation effects (71) of parsley had been studied.

Coriander (**Coriandrum sativum**) belonging to the Apiaceous family, is one of the most ancient culinary herbs known to mankind. All parts of the plant (the leaves, stems, seeds and roots), are edible and used in producing a popular spice with a pleasant lemony flavor (112). the most components in C. sativum seeds are Linalool (58.0–80.3%), α-pinene (0.2%–10.9%), γ-terpinene (0.3%–11.2%), p-cymene (0.1%–8.1%), camphor (3.0%–5.1%) and geranyl acetate (113).anti-oxidant (77, 78), food preservative (78), anti-viral (74), anti-bacterial (75) and anti-fungal(76) effects have been documented in previous studies.

Nettle (**Urtica dioica** L., Family: Urticaceae) was a well-known medicinal plant that has been used for a long time in complementary and alternative medicines worldwide (26, 81) especially in lung diseases (114). Some animal studies proposed the anti-viral effects of nettle (79, 80). Active components of garlic (**Allium sativum) **are the amino acid alliin, an alkyl derivative of cysteine alkyl sulfoxide (115). Antimicrobial activity has been studied in some researches (86). An in vitro study on the antibacterial effect of garlic (Allium sativum) on bacteria that caused wound infections, showed the crude preparation of garlic could inhibit all the tested organisms (116). Garlic showed the antiviral effects in several studies (83, 84) against different virus like Arteriviridae, Adenoviridae, Poxvirus, Flaviviridae, Coronaviridae, Orthomyxoviridae, Picornaviridae, Herpesviridae, Retroviridae etc (85). Also, an anti-oxidant and anti-cancer effect proposed (87).

Sesame oil (**Sesamum indicum) **as a supplement have anti-inflammatory (89) and antioxidant properties (88). These properties make them effective in reducing atherosclerosis and also risk of cardiovascular diseases (117). As it seems, the founded materia medica could be effective in COVID-19 as we arranged them in a hospital diet in 3 meals to be examined in a clinical trial. Details are shown in Table 2.

**Table2:** An evidence-based recommended hospital diet protocol for patients with infectious respiratory syndrome caused by COVID-19

**dinner**	**Lunch**	**Breakfast**	
Apple puree with 1 tablespoon of honey and 3 tablespoons of rose water + toasted bread + a bowl (100-200 cc) of TLS	Cooked rice with fried barberry with sugar and a little saffron + cooked chicken + some cooked carrots + a bowl (100-200 cc) of TLS	butter (20 to 30 grams) + Quince jam+ toasted bread + A small bowl (100-200 cc) Tonic lung soup (TLS^*^) + light tea with a little honey A sweet orange, one hour after breakfast	Day 1
Cooked wheat germ with and wheat flour + toasted bread + a bowl (100-200 cc) of TLS	Cooked rice with Dill+ cooked chicken + some cooked carrots + a tablespoon of concentrated juice of sweet pomegranate + A bowl (100-200 cc) of TLS	butter (20 to 30 grams) + Apple jam+ toasted bread + A small bowl (100-200 cc) TLS + light tea with a little honey A sweet orange, one hour after breakfast	Day 2
Apple puree with 1 tablespoon of honey and 3 tablespoons of rose water +toasted bread + a bowl (100-200 cc) of TLS	Cooked rice+ a meat stew (recipe: mutton + a little grated carrots, cobs and potatoes) + a little dill, parsley as a raw vegetable + A bowl (100-200 cc) of TLS	butter (20 to 30 grams) + Balang jam + toasted bread+ A small bowl (100-200 cc) TLS + light tea with a little honey A sweet orange, one hour after breakfast	Day 3
Cooked rice with Dill+ cooked chicken + some cooked carrots + a tablespoon of concentrated juice of sweet pomegranate + A bowl (100-200 cc) of TLS	One or two bowl (200-400 cc) of TLS + toasted bread Two hours after lunch: two cooked apple with sugar and saffron	butter (20 g) + Sesame cream + toasted bread + A small bowl (100-200 cc) TLS + hot saffron syrup+ rose and honey A sweet orange, one hour after breakfast	Day 4
Cooked wheat germ with and wheat flour + toasted bread + a bowl (100-200 cc) of TLS	Cooked rice with Coriander + cooked chicken + some cooked carrots + a tablespoon of concentrated juice of sweet pomegranate + A bowl (100-200 cc) of TLS	butter (20 to 30 grams) + Quince jam + toasted bread + A small bowl (100-200 cc) Tonic lung soup (TLS) + light tea with a little honey A sweet orange, one hour after breakfast	Day 5
Apple puree with 1 tablespoon of honey and 3 tablespoons of rose water + toasted bread + a bowl (100-200 cc) of TLS	Cooked rice with saffronand Raisin + cooked chicken + some cooked carrots + a tablespoon of concentrated juice of sweet pomegranate + A bowl (100-200 cc) of TLS	butter (20 to 30 grams) + Apple jam + toasted bread+ A small bowl (100-200 cc) TLS + light tea with a little honey A sweet orange, one hour after breakfast	Day 6
Cooked rice with Dill+ cooked chicken + some cooked carrots + a tablespoon of concentrated juice of sweet pomegranate + A bowl (100-200 cc) of TLS	One or two bowl (200-400 cc) of TLS + toasted bread Two hours after lunch: one cooked quince and one cooked apple with sugar and saffron	butter (20 to 30 grams) + Carrot jam+ toasted bread+ A small bowl (100-200 cc) TLS + light tea with a little honey A sweet orange, one hour after breakfast	Day 7

Notes: 

1. It is better to consume Jam without their juice at breakfast.

2. At breakfast, drink light tea instead of hot milk 

3. At lunch, the amount of meat and chicken should be controlled and most volume of food should be in TLS

4. The amount of each food item depends on the appetite of the patient.

5. Do not use spicy spices, cinnamon, pepper and ginger. Turmeric can be used in small amounts in foods.

6. It is preferred to use Sesame oil in cooking patients' food.

*Tonic Lung Soup (TLS) Recipe: A cup of wheat semolina cooked well with 5 to 6 glasses of water, a medium carrot with a clove of garlic and a small onion. If you wish, you can also add some noodles (vermicelli). Then in the last twenty minutes, add a tablespoon of chopped parsley and dill. On some days, you can add a tablespoon of dried nettle leaves to the soup. Add 25 grams of local or pasteurized butter or a tablespoon of sesame oil. Add a balanced amount of salt and turmeric. To add variety to the taste, on some days you can add a small amount of fresh lemon juice or half a lemon. Do not add meat and chicken in it.

Disclosures: Authors do not have any conflict of interest in their main file of the article. In addition, authors have the ethical responsibility to ensure all researches discussed in their work are credible and data are accurate prior to publication.
